# Women's experiences of sexual health when living with Rheumatoid Arthritis - an explorative qualitative study

**DOI:** 10.1186/1471-2474-11-240

**Published:** 2010-10-15

**Authors:** Kristina Areskoug Josefsson, Gunvor Gard

**Affiliations:** 1 Dept of Health Sciences, Health Sciences Center, Lund University, Box 157, 221 00 Lund, Sweden; 2 Samrehab, Värnamo Hospital, 331 85 Värnamo, Sweden; 3 Luleå University of Technology, Department of Health Sciences, 971 87 Luleå, Sweden

## Abstract

**Background:**

The ICF core sets for patients with Rheumatoid Arthritis (RA) acknowledge sexual function and intimate relationships as important since the patients' sexual health can be affected by the disease. About 36-70% of all RA-patients experience a reduced sexual health, and their perceived problems are directly or indirectly caused by their disease. Physiotherapy is often used as non-pharmacological treatment for RA. Mobility treatment, pain reduction, and physical activities are often included in physiotherapy for patients with RA. The aim of the study was to explore sexual health in relation to physiotherapy in women living with RA.

**Method:**

An explorative qualitative interview study with a phenomenological approach was performed. The study consisted of ten interviews with women with RA. The analysis was performed according to Giorgi.

**Results:**

The main theme that emerged in the material was that the body and the total life situation affected sexual health. Three categories were included in the theme: 1) sexual health - physical and psychological dimensions, 2) Impacts of RA, and 3) Possibilities to increase sexual health - does physiotherapy make a difference?

**Conclusions:**

Sexual health was affected by RA in different ways for the informants. Possibilities to improve sexual health were improved partner communication and physiotherapy. Physiotherapy can play an active role in improving sexual health for patients with RA.

## Background

According to World Health Organization (WHO), sexual health is a state of physical, emotional, mental and social well-being in relation to sexuality [1]. This means that sexual health has to be seen from a holistic perspective, including physical, psychological, and social aspects of well-being. To receive such a well-being also requires a positive and respectful approach to sexuality and sexual relationships.

The International Classification of Functioning, Disability and Health (ICF) [2] includes sexual health in two different areas: sexual functions and intimate relationships. The ICF core sets for patients with Rheumatoid Arthritis (RA) acknowledge both areas as important since the patients can be affected by the disease [3]. About 36-70% of all RA-patients experience reduced sexual health, and their perceived problems are directly or indirectly caused by their disease [4-8]. A majority of the patients with RA are women. However, there are differences concerning sexual health for men and women, especially during sexual activities. Women report more joint difficulties than men [9].

Sexual health difficulties due to RA can include decreased sexual arousal, decreased sexual desire, and decreased satisfaction. Different problems can cause sexual health difficulties for women with RA. It can be fatigue, pain, limited physical ability, negative body image, and depression [4,5,7,8,10-12]. Limited physical ability can affect different areas of sexual activities, for example, restricted hand mobility limits the possibility of caressing the partner [7], and restricted mobility in larger joints limits possible sexual intercourse positions [5,7,12]. According to Kraaimaat et al, there is a correlation between decreased mobility, depression, and decreased sexual health [8]. Negative body image can be connected to the amount of morning stiffness [10].

Research concerning women with RA and their sexual health has mainly focused on describing the problems, with few comments on how to improve the women's sexual health.

The mentioned solutions include increased and improved communication between health staff and patients [11] and physiotherapy. The recommendations of physiotherapy are based on pain relief treatment and joint mobility treatment with the aim to reduce pain and improve daily functional capacity as a prerequisite for engaging in sexual activities [4,8,13].

Mobility treatment, pain reduction, and physical activities are often included in physiotherapy for patients with RA. The habits of physical activity can affect sexual health, since increased physical capacity can increase the prevalence of sexual intimacy [14]. Physiotherapy treatment has been shown to increase mobility, self-esteem, and physical daily activities, and to reduce pain for patients with RA [15,16]. This could indirectly influence sexual health for this group. Concerning direct treatment of sexual health, there has been a study of cognitive-behavioural physiotherapy in order to enhance sexual function for women with chronic pain [17], which showed positive results.

## Aim

The aim of the study was to explore sexual health in relation to physiotherapy in women living with RA.

## Method

### Method and data collection

An explorative qualitative interview study with a phenomenological approach was performed. The purpose was to uncover women's experiences of sexual health when living with RA and their experiences of physiotherapy in this context. To facilitate the interviews an interview guide was used [18] that focused on experiences of sexual health when living with RA. The interview guide consisted of the following themes: marital status, duration of RA, medication, age at onset of disease, changes in the disease during the last three months, definition of sexual health, importance, experiences of sexual health over the life span and in different situations in life, relations between RA and sexual health/sexual activity/intimate relationships, suggestions for improvement of sexual health, experiences of physical and psychological functions, body image, main problems when living with RA, experiences of physiotherapy interventions, believed effect of physiotherapy, experienced emotions and usefulness of physiotherapy when living with RA. Three pilot interviews were performed before the start of the study. These were used to verify the interview guide and were not included in the study.

The informants were informed of the study by leaflets and by their local physiotherapist at the hospitals. The leaflets contained information about the study and who to contact if they wished to participate. Interested participants contacted the first author by mail or phone, and then they received additional information about the study. Before each interview all the participants received both oral and written information about the project, and confirmed their participation in the project in writing with informed consent. The project has been approved by the regional ethical committee in Linköping, Sweden. The project number is M48-09.

The individual interviews were conducted by the first author at the local hospitals. Each recorded interview lasted between 25-54 minutes, depending on the amount of information given by the informant. The interviews were transcribed verbatim. The transcribed version of the interview was sent to the participants to allow them to correct misinterpretations by the interviewer. None of the informants made any corrections of their interview material.

### Data analysis

The analysis was performed according to Giorgi [19,20]. Phenomenology was chosen since it aims to show the phenomena from the experiences and the views of the person being studied, and it is a recommended methodology for use in physiotherapy research [21,22].

Validation and reliability in phenomenology, according to Giorgi, is obtained when the described phenomena are captured by the intuited essence, and this description can be used consistently [23]. The analysis of the material according to Giorgi consists of the following steps [19,20]:

1. Reading through the material to get a general sense of the whole statement.

2. Re-reading of the material to discriminate meaning units from a holistic perspective and to focus on the experience of sexual health when living with RA.

3. Going through the meaning units and expressing deepened insight contained in them more directly.

4. Synthesizing of the transformed meaning units into a consistent statement regarding the subjects' experience.

In addition to the interviews, the informants filled in the Health Assessment Questionnaire (HAQ DI) [24] in order to ensure variability of the functional ability of the informants. The original HAQ included a question about sexual health, but the HAQ DI has excluded this question. The HAQ DI is an instrument intended to measure the informants' level of functional ability in daily activities. The HAQ DI is regularly used in rheumatology research and has been used in studies concerning sexual health and RA [5,12,25].

### Informants

Criteria for inclusion in the study were women with a diagnosed RA for at least two years, who had experience of physiotherapy, living in the county of Jönköping in Sweden. The informants should be permanent residents of the region where the study was conducted.

The 10 informants were between 42 and 66 years old, median age - 59.5 years old. A majority of the informants lived in stable, long-lasting relationships. The number of diagnosed years with RA differed between 2-31 years, median 9 years, for the informants. Some of the informants had experienced several years of joint pain before they were diagnosed. Four informants used biological medications, five had other Disease Modifying Anti-Rheumatic Drugs (DMARDs), and one informant had chosen not to have any DMARDs at all. Biological medication is treatment designed to stimulate or restore the ability of the body's immune system to fight infection and disease. This brief description of medications is to show variability among the informants concerning level of medication. The HAQ-levels of the informants varied between 0-2.13 (0 is the minimum and 3 is the maximum score possible), which indicates a great variety in daily functional ability. The work ability differed between the informants, from sickness pension to full-time employment, but most of them worked part-time. Four of the informants also had other diseases (high blood pressure, chronic obstructive lung disease and depression).

All of the informants had earlier experiences of physiotherapy treatment, to improve physical function. All of them had experience of hydrotherapy. Some of the informants had only experience from group exercises, and others had experienced both group exercises and individual physiotherapy. Group exercises included both hydrotherapy and land-based activities. The experienced individual physiotherapy consisted of: massage, acupuncture, exercise programs, stretch exercises, transcutaneous electrical nerve stimulation, and coaching towards physical activity.

## Results

The main theme that emerged in the material was that the body and the total life situation affected sexual health. Three categories were included in the theme: 1) sexual health - physical and psychological dimensions, 2) Impacts of RA and 3) Possibilities to increase sexual health - does physiotherapy make a difference?

### Sexual health - physical and psychological dimensions

Sexual health was experienced and described by the informants as containing both a physical and a psychological dimension. The physical dimension was experienced as touch, caressing, showing love and tenderness as well as sexual intercourse. The psychological dimension of sexual health was experienced as being there for another person, psychological closeness, feeling loved, and caring for someone. Some of the informants defined limits for their own sexual health, for example, explaining that sexual intercourse no longer was included in their sexual health. The informants experienced the importance of sexual intercourse differently. Sexual health was not experienced to be limited to sexual intercourse.

"Sexual health for me is not sexual intercourse. It's more like physical closeness."

Some informants had no sexual desire and were negative to sexual activities. A negative attitude towards sexual activities and loss of sexual desire affected their relationships in a negative way. The informants that described an acceptance of this by their partners or an increased acceptance over time also described less strain on their relationship. Others experienced sexual health as very important, and considered sexual intercourse as a joyful experience. All mentioned closeness and caring as important aspects of a relationship. *"Attraction, yes. That you are... That tenderness is mutual and that you...That you're now together with each other."*

The informants mentioned different emotional experiences related to their definitions of sexual health. Pleasure, happiness, joy and the opportunity to release emotions were experienced, but also increased pain and psychological pressure. Feeling attractive and being attracted to the opposite sex were also included in sexual health, as well as feelings of unattractiveness.

"You're happier simply because you've got someone."

Sexual health was also experienced as a close sexual companionship, doing things that felt good for each other, and having pleasurable sexual experiences with the partner. To achieve sexual health, understanding and communication between the partners were vital, as well as honesty with each other, and willingness to participate in the sexual relationship. Sexual health and the importance of sexual health were experienced to have changed in different phases of life.

"Yes. I don't put too much weight in it. It was more important when I was younger. One does revalue things. I wouldn't have responded like this 10 years ago."

### Impacts of RA

The informants experienced that RA could have physical, psychological, emotional, and relational impacts on them. Most informants experienced negative impacts of RA, both directly and indirectly, on their sexual health.

Physical impacts of RA were experienced to be similar for the informants such as pain, fatigue, stiffness, and swollen joints, with varying severity. Some of the informants also experienced decreased physical ability, deformed joints, and reduced muscle strength, which could lead to decreased ability to walk and to perform daily activities. There was a large variety of direct negative impact on sexual health: deformed hip joints which made sexual intercourse impossible, pain (before, during and after sexual activities), hip mobility problems during intercourse, decreased mobility in sexual activities in caresses, decreased sexual arousal and desire, pain when being touched, pain when caressing the partner, decreased sexual satisfaction, and fewer possible positions for sexual intercourse. Caresses could lead to increased pain, and in those cases further sexual activities were often avoided.

"And then, it hurts so much when someone touches me that I feel, no, you mustn't touch me."

"I've lost my sexual desire."

"Sex simply doesn't work. I can barely walk sometimes."

Some of the informants had gone through or planned to go through joint surgery.

Body image was experienced to be affected for some of the informants, and the most common negative impact was increased weight due to medication and inability to be physically active. RA was experienced to influence the whole life situation.

Psychological impacts of RA were experienced but differed within the group. A positive impact was increased psychological strength, which was achieved by having to handle the physical impacts of RA. Negative impacts were problems due to sleeping difficulties, anxiety, worry, frustration, fear of being abandoned by their partner, feeling old, and being angry. The negative emotions affected the sexual life of the informants, for example, worrying about increased pain during sexual activities led to avoidance of sexual activities, and feeling old was connected to feelings of being unattractive.

"...but I get so damned mad for I can't manage anything without feeling pain as a result of it."

"For I have felt I'm not good enough. In different ways, not just sexually."

The relational impacts of RA were experienced within the field of sexuality, as for example different sexual needs. Other relational impacts of RA concerned reduced capacity to perform daily activities. The informants described experiences both from present and earlier relationships. One informant experienced an improved relationship with her partner after the RA diagnosis.

"Well, it's a little like this - what I can do and what I can't do. The discussion pops up now and then on what we want to do. He wants to do it, but I can't and that's a pity, I think."

"I couldn't manage anything. I couldn't even manage intimacy because I couldn't bear being touched."

### Possibilities to increase sexual health - does physiotherapy make a difference?

Reflections about possibilities to improve sexual health were a new field for the informants. Most of them had not reflected on their sexual health or thought it possible to improve it. Some informants were satisfied with their sexual life. However, in the interview situations, possibilities to increase sexual health were explored. The informants described that if their pain and fatigue could be decreased, and if their sexual desire could be increased, they believed that their sexual health would be improved.

"If I hadn't had pain, it would have worked better. Take away tiredness."

Some of the informants had experienced direct positive effect of physiotherapy on their sexual health, and some had not reflected on this topic before the interviews. Several of the informants described perceived effects of physiotherapy for rheumatic patients in general, and not only from their personal experiences.

Direct experienced effects of physiotherapy concerning sexual health included increased physical activity levels, knowledge of their own physical capacities, and increased fantasy. Increased knowledge of physical abilities and increased physical capacity could make sexual activities easier since the informants felt that they had enough physical strength to manage specific intercourse positions. Physical activities during physiotherapy were described as a way to get a widened range of positions that could be used in sexual activities. Touch and increased ability to accept being touched were mentioned by some informants as important for sexual health. Several physiotherapy interventions include touching and passive joint mobility stretching. Increased joint movement was given as a suggestion for more possible positions for sexual intercourse. Increased relaxation was mentioned as an effect of physiotherapy that improved sexual health.

"I think it has helped me to be more agile."

"Because you get softer in your body. You get energy as well. You get a burst of energy."

The positive emotions following a physiotherapy session were experienced to have given an increased attention for the partner and more energy to the relationship. Increased self-esteem and a positive body image were experienced to have improved the feeling of sexual attractiveness.

"Yes, I think so. In any case, to maintain agility and strength and to feel satisfied. You're in fact exercising and you feel satisfied with your body."

Some of the informants believed physiotherapy to be more important concerning sexual health for patients with severely decreased mobility and low range of joint movement. Exercise programs that decreased pain were also believed to improve sexual health, since several of the informants mentioned pain as an important factor that could decrease sexual health.

Informants experienced both positive and negative effects of physiotherapy, and all of the informants still had a positive opinion of physiotherapy, and had experienced emotions of contentment and joy regarding treatment sessions. All informants expressed a flow of positive emotions during and after physiotherapy, such as joy, feeling appreciated and cared for. These emotions increased the experienced positive effects of physiotherapy.

The informants described their physiotherapists as a coach, a provider of information concerning the body and the disease, someone who gave them feedback and back-up in their daily activities. The physiotherapist was considered by the informants to have an important role in rheumatological care.

"It's also that you get... how you should do and feedback and I ask like this: 'What should I do if I want to have muscles there? If I want help with the right exercises."

According to the informants, sexual health could also be improved by imagination, fantasies, and planning of sexual activities that consisted of: spending time together on holidays, creating a more intimate atmosphere with your partner, finding time for more foreplay and using stimuli for love, such as music.

An important aspect to achieving sexual health was an increased communication between partners and knowledge about the impacts of RA concerning sexual health. Written information, shared with the partner, could be used as a basis to discuss their own mutual sexual life. Long-lasting relationships were experienced to increase the possibilities of having an open communication concerning sexual health between partners. The partner's own health also affected the sexual health for the informants, and a healthy spouse increased the possibilities of having good sexual health.

## Discussion

As this is an explorative first study of experiences of sexual health in relation to physiotherapy, we used a qualitative design with a phenomenological approach to cover the lived experiences of sexual health when living with RA. Phenomenological approach with analysis according to Giorgi, has been used in earlier physiotherapy research concerning musculoskeletal disorders [26] and in other studies in rheumatology. One of the interviews was short time-wise, which could indicate that the researched phenomena were not fully explored [18], and it is possible that the informant was not willing to share her views of the researched phenomena due to the sensitivity of the topic. A follow-up interview with this informant might have given additional data. The phenomenological research approach indicates that a small sample size is sufficient to explore the researched phenomena [27] and to ensure that the collected data can be fully explored. There has been discussion concerning the truthfulness of face-to-face interviews in sexuality research [28], but as the first exploratory study of experiences of women's sexual health when living with RA and their experiences of physiotherapy in this context, phenomenology was chosen as the most appropriate method for the study.

The informants were informed that the interviewer was a physiotherapist, which might have influenced the believed effects of physiotherapy in a positive way. It is also possible that the knowledge of the interviewer, concerning physiotherapy for RA, increased the ability to deepen the interviews. Counter effects of medicines were not brought up in the interviews since they are not considered relevant from a physiotherapy perspective, even if those effects might affect sexual health. The revealed information in this area is limited to what the informants included in the perspective of sexual health. The emerged themes are specific to the informants and the context, but generalisation could be possible to other individuals in similar contexts.

Changes in health can lead to reducing the importance of sex in life [29], and results showed that multidimensional factors, due to RA, influenced sexual health for the informants.

The results showed that decreased mobility and muscle strength could have a negative impact on sexual health due to difficulty in finding comfortable positions during sexual activities, or limiting the amount of possible sexual intercourse positions. Those findings coincide with findings in other research [4,8,9,11,12,30]. Inability to be as physically active as the informants wanted to be was mentioned as a reason for reduced sexual health. This could be important knowledge in clinical practice since physiotherapy often is directed towards increasing physical activity. Body image did not seem to be a major issue for the informants, even though some of them were visually marked by RA. The reasons for this could be a field for future research.

Emotional experiences due to RA were anger, frustration, and fear of being abandoned by the partner. Fear of being left by the partner can depend on decreased sexual activities [31]. Those emotions can affect sexual health in a negative way, and might need to be addressed more often when talking about sexual health for patients with RA.

The informants mentioned not only pain in connection to sexual intercourse, but also pain when being touched/caressed, and this needs to be considered since caresses are still important in a relationship, when the importance of sexual intercourse is lowered. Pain when being caressed has not been focused on in other studies.

Changes during life and age can affect sexual desire [32], and age also has an effect on the importance of sexual ability for patients with RA [11], which implies that the interviews might have differed concerning importance of sexual health and sexual activities, if the informants had been younger. The age of the informants indicates that several of them should have experienced menopause, but none of them mentioned this in the interviews. This is interesting since menopause and hormonal changes can affect both RA [33,34] and sexual health [35].

Decreased sexual satisfaction was mentioned by some of the informants. This has been brought up in other studies, but the conclusions differ [9,12,36,37]. According to van Lankveld et al, sexual satisfaction is lower among patients with RA and their partners [36] compared to healthy individuals. This was unrelated to pain and level of functional ability. Majerovitz and Revenson [37] disagree with those results, and indicate that greater functional disability is related to lower sexual satisfaction, especially for women. Van Berlo's findings show no differences in sexual satisfaction between patients with RA and controls [9].

Decreased sexual health is common for women with RA, and their sexual health problems remain after two years of RA, despite medication [38]. This shows the importance of finding ways to improve sexual health for women with RA. The most common areas mentioned for improvement by the informants were decrease of pain and fatigue, as well as increase of sexual arousal. Reflections on how to improve sexual health were quite new to the informants, and it is possible that other problems related to RA were more prioritized. Practical suggestions on how to improve sexual health included better communication between partners, better information about consequences of RA on sexual health, and physiotherapy.

Physiotherapy can affect sexual health positively in different areas according to the informants. According to Hill et al [11] most of the sexual health problems due to RA are related to symptoms which could explain why the informants thought or had experienced that physiotherapy could improve sexual health. Physiotherapy is often directed towards improving activities of daily living, and sexual relations should be included in this context [39]. Physiotherapy in general has changed towards becoming more of an active treatment, including coaching towards increased physical activities [40]. Increased activity levels can increase perceived health status and reduce fatigue for patients with RA [41,42], and were mentioned as giving direct positive effect on sexual health by the informants, which is also stated by Monga et al. [43]. Those results coincide with results of older healthy women, where physical fitness and high levels of sexual activity were related [14]. Physiotherapy interventions based on exercise therapy can also reduce fatigue [42], which then indirectly can increase sexual health according to the informants. Pain was decreased by physiotherapy, and since pain is one of the dominating factors that decrease sexual health, physiotherapy must be highlighted as an important way to improve sexual health for patients with RA. Increased muscle relaxation was mentioned as a positive effect of physiotherapy by the informants, but progressive muscle relaxation interventions on quality of life for patients with RA have not been shown to have long-term effect [44]. Physiotherapy was considered to increase the amount of possible sexual intercourse positions by increasing joint mobility, increased muscle strength, and the patients' knowledge of their own physical abilities. Muscle strength exercise is common treatment for patients with RA [45], and is usually supervised by a physiotherapist. Knowledge of one's own physical ability was connected to positive self-esteem and a more positive view of the body, and increased feeling of sexual attractiveness, all of which can influence sexual health. Positive emotional reactions during physiotherapy were mentioned by the informants, and those emotions can increase the effect of physiotherapy [46]. There were also positive emotional reactions after physiotherapy, which the informants noted as positive for their relationship with their partner. Some of the informants believed that physiotherapy gave a greater effect on sexual health for patients with more severe physical limitations. As authors we believe that physiotherapy has an important role in improving sexual health for women with RA. The study has deepened the knowledge of experiences of sexual health for women with RA.

The awareness of physiotherapists concerning the possibilities of affecting sexual health directly/indirectly for women with RA is a field for future research, as well as whether/how women with RA want physiotherapists to address with them the issue of sexual health in relation with physiotherapy. There is a need for further research concerning whether physiotherapy treatment directed at improving sexual health for women with RA can get positive results, or whether effect of physiotherapy on sexual health only can be seen as an indirect co-effect of physiotherapy interventions in general. There also is a need for research concerning which physiotherapy interventions are most effective to use in order to improve sexual health, in order to evaluate direct effect of physiotherapy on sexual health.

## Conclusion

• Sexual health was affected by RA in different ways for the informants.

• Possibilities to improve sexual health were improved partner communication and physiotherapy.

• Physiotherapy can play an active role in improving sexual health for patients with RA.

## Competing interests

The authors declare that they have no competing interests.

## Authors' contributions

The planning of the study, data analysis, results, and discussion were performed by both authors. KAJ performed the data collection and drafted the section on results and discussion. Both authors read and approved the final manuscript.

## Pre-publication history

The pre-publication history for this paper can be accessed here:

http://www.biomedcentral.com/1471-2474/11/240/prepub
